# Cholera

**Published:** 1848-03

**Authors:** 


*Cholera.*—The French government has appointed three physicians to proceed to Russia, to study the cause of the cholera. These physicians are Drs. Bean, Monneret, and Coutour, and they will take up their stations at Moscow, Odessa, and Trebizond. The last city is in a great measure deserted; instead of containing 60,000 inhabitants, but 10,000 remain in it.—*Ib.*

---

*Chronic Enlargement of the Tonsils.*—Dr. Naudin, instead of producing a slow, progressive destruction of the tonsils, aims at their preservation, and for this purpose employs a solution of nitrate of silver, three grains to one ounce of water, increasing the strength by three grains up to two drachms of the nitrate, in the same quantity of water, and also applying the solid caustic to the surface of those hollows which usually exist in such tonsils, so that all parts may be equally affected. During one sitting the tonsils are painted twice or thrice; the mouth is then well washed with water. This cauterization must be repeated every two or three weeks until the tonsils are restored to their normal size, and then gradually discontinued; it produces no ill consequences, and even children speedily return to their play. Should the parts become accustomed to the caustic, it must either be discontinued for a time, or another substituted, as Lugol's diluted solution of iodine. In two cases related by our author, the nitrate alone was employed. Both, aged thirteen and fourteen, had been affected for years, and were cured in two and a half to three months; in a third case—that of a girl aged eleven—the disease was extensive and obstinate, requiring four months' use of the caustic, besides the use of hyd. pot. and iodine internally, and as an ointment. In all these cases no return has been observed after the lapse of years, and the previous disposition to inflammation of the tonsils has been extinguished.—*Edinburgh Monthly Journal.*

